# Editorial: New Development of Tracers Uses in Gynecologic Surgery

**DOI:** 10.3389/fonc.2022.912267

**Published:** 2022-05-31

**Authors:** Stefano Cianci, Salvatore Gueli Alletti, Francesco Fanfani

**Affiliations:** ^1^ Department of Human Pathology of Adult and Childhood “G. Barresi” Unit of Gynecology and Obstetric, University of Messina, Messina, Italy; ^2^ Division of Gynecologic Oncology, Fondazione Policlinico Universitario A. Gemelli Istituto di Ricovero e Cura a Carattere Scientifico (IRCCS), Rome, Italy; ^3^ Institute of Obstetrics and Gynecology, Universita’ Cattolica del Sacro Cuore, Rome, Italy

**Keywords:** gynecologic oncology, fluorescence, indocyanine green, gynecologic surgery, novel tracers

Intra-operatory fluorescence imaging represents a fundamental step for many surgeries. The possibility to identify anatomical structures, healthy and non-healthy tissues, and vascular perfusion of different structures represent a cornerstone of a modern surgery not only for gynecology but for all abdominal surgeries (1, 2).

The fluorescence imaging allows to improve the surgical specificity reducing the impact of surgical invasiveness with the aim to reduce the complications and patient’s quality of life (3, 4).

The most recent innovation in tracers is represented by indocyanine green (ICG), a cyanine dye tracer detected by the use of near infrared imaging technologies, have emerged as feasible alternatives to the traditional methods as blue dyes and radiolabeled tracers (5).

This new technology is most applied in gynecologic surgery, in particular for endometrial cancer and cervical cancer sentinel lymph-node mapping. Recently its application has been extended to ovarian cancer lymph-nodal mapping (6, 7). It has also used even for benign pathology for endometriosis detection (8). Other applications are represented by the use of tracer to verify the blood perfusion of tissues and anatomical structures after surgery (2).

It was an honor and a pleasure for us to serve as Guest Editors of the Research Topic of *Frontiers* entitled “New Development of Tracers Uses in Gynecologic Surgery”. We are pleased to present a series of articles produced by proven experts in the field of gynecology. All authors that contributed to the Research Topic are authors contributing to important advancements in clinical and basic research. This Research Topic provides an overview about the tracers use in gynecology and their integration in clinical practice.

We believe that the topics deepened in this Research Topic will be of interest to a wide audience, from basic academic researchers to clinicians in gynecology, oncologists, and surgeons.

The Research Topic opens with a randomized trial by Alletti et al. entitled “A Multicentric Randomized Trial to Evaluate the Role of Uterine Manipulator on Laparoscopic/Robotic Hysterectomy for the Treatment of Early-Stage Endometrial Cancer: The ROMANHY Trial.” reporting the integration and the outcomes of sentinel lymph node for endometrial cancer treatment.

The second article by Bizzarri et al. entitled “Indocyanine Green to Assess Vascularity of Ileal Conduit Anastomosis During Pelvic Exenteration for Recurrent/Persistent Gynecological Cancer: A Pilot Study” evaluating the use of indocyanine green to improve the surgical outcomes after major surgery. Especially reporting the advantages of tracers use to detect eventual vascular defect of anastomosis for prevention of bowel dehiscence.

The third article by Li et al. entitled “Fertility outcome and safety of ethiodized poppy seed oil for hysterosalpingography in 1053 infertile patients: a real-world study” evaluating and comparing the use of a different contrasts for hysterosalpoingography on infertile patients.

The fourth article by Cianci et al. entitled “Sentinel Lymph Node in Aged Endometrial Cancer Patients the SAGE Study: A Multicenter Experience” focused on the sentinel lymphnode detection in elderly patients and aimed to evaluate the detection rate and the surgical outcomes of this selected subset.

The last article by Capozzi et al. entitled “Subcutaneous Vulvar Flap Viability Evaluation With Near-Infrared Probe and Indocyanine Green for Vulvar Cancer Reconstructive Surgery: A Feasible Technique” evaluating the use of indocyanine green for vulvar cancer reconstructive surgery with the aim to identify and define the vascularization of flaps and the surgical outcomes.

We would like to thank all authors for these fine and interesting articles which makes a significant contribution on scientific panorama.

## Author Contributions

SC, SGA, FF: writing, literature search. All authors: reviewing of the final manuscript. All authors contributed to the article and approved the submitted version

## Conflict of Interest

The authors declare that the research was conducted in the absence of any commercial or financial relationships that could be construed as a potential conflict of interest.

## Publisher’s Note

All claims expressed in this article are solely those of the authors and do not necessarily represent those of their affiliated organizations, or those of the publisher, the editors and the reviewers. Any product that may be evaluated in this article, or claim that may be made by its manufacturer, is not guaranteed or endorsed by the publisher.
